# Validation and Predictive Utility of a Person-Centered Quality of Contraceptive Counseling (QCC-10) Scale in Sub-Saharan Africa: A Multicountry Study of Family Planning Clients and a New Indicator for Measuring High-Quality, Rights-Based Care

**DOI:** 10.1111/sifp.12229

**Published:** 2023-02-14

**Authors:** Celia Karp, Funmilola M. OlaOlorun, Georges Guiella, Peter Gichangi, Yoonjoung Choi, Philip Anglewicz, Kelsey Holt

**Affiliations:** Department of Population, Family and Reproductive Health, Johns Hopkins Bloomberg School of Public Health, Baltimore, MD, 21205, USA.; Department of Community Medicine, College of Medicine, University of Ibadan, Ibadan, Nigeria.; Institut Supérieur des Sciences de la Population (ISSP/University Joseph Ki-Zerbo), Ouagadougou, Burkina Faso.; International Centre for Reproductive Health-Kenya, Nairobi, Kenya.; iSquared, Severna Park, MD, USA.; Department of Population, Family and Reproductive Health, Johns Hopkins Bloomberg School of Public Health, Baltimore, MD, 21205, USA.; Department of Family & Community Medicine, University of California, San Francisco, CA, 94110, USA.

## Abstract

The lack of validated, cross-cultural measures for examining quality of contraceptive counseling compromises progress toward improved services. We tested the validity and reliability of the 10-item Quality of Contraceptive Counseling scale (QCC-10) and its association with continued protection from unintended pregnancy and person-centered outcomes using longitudinal data from women aged 15–49 in Burkina Faso, Kenya, andNigeria. Psychometric analysis showed moderate-to-strong reliability (alphas: 0.73–0.91) and high convergent validity with greatest service satisfaction. At follow-up, QCC-10 scores were not associated with continued pregnancy protection but were linked to contraceptive informational needs being met among Burkinabe and Kenyan women; the reverse was true in Kano. Higher QCC-10 scores were also associated with care-seeking among Kenyan women experiencing side effects. The QCC-10 is a validated scale for assessing quality of contraceptive counseling across diverse contexts. Future work is needed to improve understanding of how the QCC-10 relates to person-centered measures of reproductive health.

## BACKGROUND

High-quality contraceptive services are essential for ensuring human rights in reproductive healthcare. While service quality has long been a focus in family planning (Bruce 1990; Tumlinson 2016), its measurement has proved challenging. Quality of contraceptive care is fundamentally different from other clinical areas, such as maternal health, due to the centrality of the counseling experience itself, rather than a medical intervention, and the fact that contraceptive choice is a preference-sensitive decision without one best outcome for all people (Dehlendorf et al. 2017). Researchers continue to grapple with a lack of agreement about standardized quality measures, particularly related to contraceptive counseling—the corner-stone of client–provider interactions in family planning (Tumlinson 2016; RamaRao and Jain 2016; RamaRao and Mohanam 2003). Variability in context-specific definitions of a “positive” counseling experience further complicates measurement (Holt et al. 2018; Dehlendorf et al. 2013). Challenges also arise with the application of quality metrics originating in high-income countries to low- and middle-income country settings, where their relevance may be limited. Without systematic evaluation and psychometric assessment of quality indicators in new geographies, distinctions in the conceptualization of quality may inhibit utility of these metrics. Existing indicators also skew toward positive aspects of care, often neglecting negative experiences (e.g., biased counseling) that are critical to measure for ensuring the fulfillment of human rights in family planning services globally (Reichenbach, Hardee, and Harris 2016; Senderowicz 2019; Solo 2019).

Researchers have historically applied Judith Bruce’s quality of care framework to investigate the quality of contraceptive counseling, with most measures focused on technical aspects of care, including Bruce’s element of “information given to clients” (Bruce 1990). Today, programs rely heavily on the Method Information Index (MII) and the Method Information Index Plus (MII+), an indicator for which clients are asked to report on their receipt of specific counseling messages (i.e., told about multiple methods, side effects, what to do if you experience side effects, and option to switch methods). Measures like the MII+ have proved critical for identifying gaps in the delivery of comprehensive information during contraceptive services (Jain, Aruldas, Tobey, et al. 2019; Chang et al. 2019; Chakraborty et al. 2019). Despite their utility, however, such measures are limited in capturing the full range of what constitutes a high-quality, person-centered counseling experience. To measure information provision, the MII+ uses a predefined list of topics the provider is expected to cover. Notably lacking are items related to interpersonal treatment and respect for informed choice (Senderowicz 2020; Bullington et al. 2022), thereby excluding assessment of the degree to which individuals, themselves, deemed their informational needs were met. The measurement of this alignment between services and individuals’ preferences is critical given the wide range of possible needs during contraceptive counseling (Kennedy, Yeh, and Gaffield 2021).

Increasing recognition of the need for person-centered, rights-based care has resulted in new frameworks and measurement tools that explicitly center human rights in contraceptive services. An emphasis on person-centered care extends the measurement of quality in contraceptive counseling beyond clinical dimensions to encompass aspects like supportive and respectful care and autonomy in contraceptive choices. In 2017, Holt and colleagues proposed a framework to conceptualize dimensions of high-quality, right-based contraceptive counseling, outlining three dimensions of care, including assessment of needs and preferences; provision of neutral, unbiased, and information-centered decision-making support; and respect for method choice (Holt, Dehlendorf, and Langer 2017). This framework built on Bruce’s framework by detailing specific elements of the counseling process that are critical to person-centered, human rights-based service delivery.

Recent developments in our understanding of quality and person-centeredness in family planning have also led to advances in measurement of this concept, including several instruments ranging in scope, length, and origin. In the past decade, two important additions to the compendium of measures were developed, including the Interpersonal Quality of Family Planning (IQFP) and the Person-Centered Family Planning (PCFP) scales. In 2018, the IQFP was developed from formative, qualitative research in the United States to reflect quality domains, including receipt of adequate information, interpersonal connection, and decision support (Dehlendorf et al. 2016, 2018); the tool was later validated among women in one Indian state (Johns et al. 2020). With its emphasis on examining client preferences, needs, and values in their counseling sessions, the IQFP filled an important gap between technical elements of care and person-centered perspectives about what characterizes high-quality care in family planning. Around this time, Sudhinaraset and colleagues developed the PCFP scale in India and Kenya, consisting of 20–22 items, varying by context (Sudhinaraset et al. 2018). Items in the PCFP tool were grounded in the literature, identified from existing measures of quality in maternal and reproductive care, and refined based on expert review and cognitive testing. The PCFP comprises two subscales capturing domains of process and structural quality, including autonomy, respectful care, and communication; and the health facility environment; the predictive utility of the PCFP for reproductive health outcomes has yet to be assessed. Together, the IQFP and PCFP helped pave the way toward improved metrics of quality in family planning, offering new prospects for the evaluation and improvement of quality care. Despite progress, however, measurement gaps remain, including a lack of counseling-specific measurement that is grounded in human rights and captures negative experiences of care.

The Quality of Contraceptive Counseling (QCC) scale expands on Holt et al.’s framework, establishing a measure that reflects a person-centered approach to care and was developed through formative research across three continents in the Global South (Holt et al. 2019). The 22-item QCC scale, including three subscales covering domains of *information exchange*, *interpersonal relations*, and *disrespect and abuse*, extends beyond technical aspects of care to ascertain individuals’ experience of care. The scale was validated in Mexico and later refined in Ethiopia and India (Holt, Uttekar, et al. 2021; Holt et al., n.d.). In its initial development, the QCC scale was found to be positively and significantly associated with women reporting their informational needs were met within the first three months of their family planning visit, measured via a single item that asked women if they needed more information about contraceptive methods (yes/no) (Holt et al. 2019). Joining the IQFP and PCFP, the QCC became one of the first instruments generated from formative qualitative interviews in low- and middle-income countries to measure quality, person-centeredness, and negative elements of care in family planning.

Recognizing the need for a shorter measure in contexts or existing surveys where 22 items may be infeasible, the QCC scale was optimized and reduced to a 10-item measure, using data from Ethiopia, India, and Mexico (Holt, Karp, et al. 2021). The resulting QCC-10 is a unidimensional adaptation of the QCC scale, comprising items reflecting the original three subscale domains, each representing important aspects of process quality and the direct elicitation of client needs being met in counseling. The QCC-10 holds great promise for systematic measurement of quality and person-centeredness in family planning services, but it has yet to be evaluated in a broader range of contexts or assessed in relation to reproductive health outcomes over time.

Our research team, including members of the original QCC scale development group, sought to address these gaps in available, cross-cultural measures of quality and person-centeredness in family planning by testing the QCC-10 in new geographies. The objectives of this study were to measure the validity of the QCC-10 scale in four sub-Saharan African geographies in three culturally diverse countries and examine the predictive utility of the QCC-10 for understanding a range of person-centered measures of reproductive health six months after contraceptive care was received.

## STUDY SETTINGS

This study takes place in four geographies in sub-Saharan Africa, including Burkina Faso, Kenya, and two states in Nigeria (Kano and Lagos). The reproductive health contexts of these countries differ, with estimated total fertility rates spanning from 3.4 children per woman of reproductive age in Lagos and Kenya to 5.5 in Burkina Faso and 6.5 in Kano (World Bank 2019; National Population Commission (NPC) [Nigeria] and ICF International 2019). Access to, desire for, and use of family planning services also vary widely across contexts. Modern contraceptive prevalence rates range from 12 percent among women in Nigeria (8 percent in Kano and 24 percent in Lagos) to 28 percent in Burkina Faso and 42 percent in Kenya (Centre for Research Evaluation Resources and Development 2021a, 2021b; Track20 2021a, 2021b, 2021c). Intention to use contraception mirrors these trends in use, with low proportions of contraceptive nonusers intending to adopt a method in the next year in Lagos and Kano (7 percent and 11 percent, respectively), relative to more than one-quarter of nonusers who plan to start a method in Burkina Faso and Kenya (Centre for Research Evaluation Resources and Development 2021a, 2021b; l’Institut Supérieur des Sciences de la Population at l’Université Joseph Ki-Zerbo 2021; International Centre for Reproductive Health Kenya 2021). Injectables and implants are the most widely used methods among married women across geographies, with the exception of Lagos where male condoms are reported by one-quarter of married women. In terms of contraceptive counseling, receipt of the four informational MII+ components ranges from 29 percent in Lagos to 60 percent in Kano. The rich diversity of these geographies makes them the ideal settings for exploring the transfer-ability of the QCC-10 to new contexts beyond Ethiopia, India, and Mexico, where the scale originated.

## METHODS

### Data

We used longitudinal panel data from women who received family planning services in Burkina Faso, Kenya, and Nigeria (Kano and Lagos states) in 2020–2021, amid the COVID-19 pandemic. Data were collected as part of the Performance Monitoring for Action (PMA) project, which implements nationally or regionally representative household surveys of women aged 15–49 to generate data on a range of key reproductive health indicators, including use of family planning (Zimmerman et al. 2017). Women were recruited for participation as part of PMA’s client exit interviews from a sample of public and private health facilities serving the selected enumeration areas where PMA’s household surveys were implemented. The 10-item QCC-10 scale was added to the client exit interview, alongside standard survey questions that asked women about their care experience. Further information about the PMA survey design and sampling strategy have been published elsewhere and can be found at www.pmadata.org (Zimmerman et al. 2017).

In each geography, client exit interviews were conducted in sampled facilities where monthly client family planning (FP) caseloads were at least three FP clients per day on average, considered “moderate” caseloads (caseload calculation = total FP clients [minus condom clients] served in the past month divided by the number of days in a month facility provides FP services; rounded to the nearest whole number). Interviews were conducted in each facility over a two-day period. As with other PMA surveys, female interviewers resided within or near the community where data collection took place. Interviewers conducted all surveys in a private location within or nearby the health facility. After seeking approval from health facility directors, interviewers recruited women into the study, screened them for eligibility, and conducted informed consent processes. Women were eligible to participate if they were aged 15–49 and had received information about family planning at the facility on the day of interview, regardless of if they received contraception, were prescribed a method, or neither.

Baseline interviews were conducted face-to-face and lasted approximately 30–60 minutes. Women were asked about their sociodemographic and reproductive characteristics, experience at the facility, perception of the care they received, and other information about their family planning visit on the day of the interview. As this was the first use of the QCC-10 scale in these four sub-Saharan African geographies, translating and assessing comprehension of scale items was a critical first step prior to survey implementation. Translation and cognitive testing procedures followed standard PMA practice for use of new survey items. Scale items were translated into all local languages by PMA survey teams and then pilot-tested for cognitive understanding among women in each geography. Final translated items were then integrated into the survey, following introductory language for interviewers to explain the agreement scale options. During the COVID-19 pandemic, all face-to-face surveys were administered with appropriate social distancing and other safety protocols (e.g., mask-wearing), as necessary.

Phone-based follow-up surveys were conducted approximately 4–6 months after their initial counseling visit among women who received a contraceptive method or prescription for a method at their baseline family planning visit (the vast majority of women surveyed at baseline). Among eligible women, those who were willing to participate in a follow-up survey and had access to a phone were recontacted for follow-up. Nearly all women who were successfully contacted agreed to participate and completed the follow-up survey (Online Appendix Table T1); follow-up interviews lasted approximately 15–30 minutes. Phone-based follow-up eligibility and survey completion rates varied by geography, ranging from 87.6 percent and 95.4 percent of women who were eligible for follow-up in Burkina Faso and Kano, respectively, to 85.0 percent and 72.2 percent of eligible women ultimately completing the follow-up interview in Kano and Kenya, respectively.

### Measures

Our primary outcome was quality of contraceptive counseling, measured via the QCC-10 scale, presented in Table 1. Items in the QCC-10 map to each of the three domains of quality in contraceptive services, including *information exchange* (five items), *interpersonal relations* (three items), and *disrespect and abuse* (two items). Participants in the baseline survey were asked to indicate their level of agreement with each QCC-10 scale item using a four-point Likert scale. Response categories included “completely agree,” “agree,” “disagree,” and “completely disagree” for each item. Participants who were unsure of their answer could indicate “do not know” or refuse a response to the item. Each agreement response was coded on a scale of one to four, with higher scores indicating higher quality counseling. For example, for positively worded items like, “I felt encouraged to ask questions and express my concerns” (item: express_self), responses of “completely agree” were coded as 4 and “completely disagree” as 1. Two items capturing *disrespect and abuse* probed for negative counseling experiences (e.g., biased counseling based on women’s characteristics and provider preferences); responses were reverse coded for these two items (items: prov_insist, scold_marital). Mean scores were computed for the composite 10-item QCC-10 scale as the average of all items (range: 1–4). Exact item wording for several items varied slightly from the original QCC-10 development, given adjustments made by the PMA research teams to accommodate in-country preferences.

At baseline, we measured client satisfaction by asking women how satisfied they were with the family planning services they received. We selected a global measure of client visit satisfaction for our initial assessment of the QCC-10, as service satisfaction relates to whether individuals receive person-centered care but is also recognized as a distinct aspect of individual’s perceptions of care. Widely used satisfaction measures are grounded in expectation disconfirmation theory and, thus, are often a reflection of how people’s care experiences aligned with the care they expected, instead of how aligned care was with their individual needs (Kupfer and Bond 2012; Brien 2009). While the QCC-10 items and overall scale score are more specific than this global measure, we hypothesized that client reporting of greatest satisfaction with family planning services would be associated with higher QCC-10 scores. Responses to the client satisfaction item were captured with a five-point Likert scale, ranging from “very satisfied” to “very dissatisfied.” Exploratory analysis, sample size considerations, and courtesy bias in facility-based client exit interviews resulted in use of a top-scoring approach, dichotomizing responses to distinguish women who reported being “very satisfied” (herein referred to as “high satisfaction”) from other reported experiences.

At follow-up, we sought to examine relationships between higher quality contraceptive counseling at baseline and reproductive health measures within the first six months after-care. Beyond recent advances in the measurement of quality itself, efforts to understand the implications of receiving high-quality contraceptive counseling have been challenged by the identification of appropriate person-centered outcomes. For example, several quality measures have been evaluated according to the measure’s ability to predict whether women continue to use their method or any method. While contraceptive continuation provides insight into individuals’ contraceptive trajectories following care, reliance on this measure is not person-centered, as it neglects the fact that many people discontinue for reasons that align with their preferences, including a desire or ambivalence towards becoming pregnant (Senderowicz 2020). Innovations are needed to identify and test relevant person-centered outcome measures for understanding long-term impacts of high-quality contraceptive care. We used three reproductive health measures available in the follow-up survey, including two person-centered measures that approximated the degree to which individuals’ contraceptive needs were met. First, we assessed whether women experienced continued protection from unintended pregnancy. Next, focusing on person-centered measures, we examined whether women’s informational needs were met at follow-up, and, among those who experienced side effects, whether they sought care from a health provider to help manage their side effects.

We assessed women’s continued protection from unintended pregnancy at follow-up by asking a series of questions about their reproductive status and contraceptive practices since the initial counseling visit, assuming that women who were seeking contraceptive services at baseline desired to avoid pregnancy in the near-term (Online Appendix Figure A1). We asked women if they were pregnant, had started and were still using the contraceptive method prescribed or provided at baseline, had switched or adopted a new method, had discontinued contraception altogether, and if so, their reasons for discontinuation, and if they experienced side effects related to their contraceptive method(s). We categorized women as experiencing an “unintended” pregnancy if women were pregnant at follow-up and reported contraceptive method failure or reported discontinuing their method due to low perceived risk (i.e., “Infrequent sex/husband/partner away” or “Difficult to get pregnant/menopausal”).

Similarly, we categorized women as “discontinuing while still at risk of unintended pregnancy” if they stopped using their method for reasons other than a perceived low risk of pregnancy or wanted to/became pregnant (i.e., intended pregnancy). Women were considered to have continued protection from unintended pregnancy since their initial counseling visit if they did not experience an unintended pregnancy nor discontinue their method while still at risk of pregnancy (Table 5).

Our first person-centered measure explored the extent to which women’s informational needs about contraception were met at follow-up through four questions that aligned with components of the MII+. We asked women to consider their current perceptions of the information they received at their initial counseling visit, specifically, “Do you feel you received too much, too little, or just enough information about (a) side effects that you might experience, (b) what to do if you experience problems, (c) how to switch methods, and (d) how to stop using your method?” For each component, we coded women’s responses of “too little” as 0, “too much” as 1, and “just enough” as 2, and then calculated summative scores to assess how comprehensively women’s informational needs were met at follow-up through a simple additive score (range: 0–8). We used a continuous score to capture nuance about how useful the quantity of the information ultimately was in helping women navigate and manage their health needs related to their contraceptive use. Similarly, we coded responses of “too much” as distinct from “just enough” because we focused on person-centered measures that reflected alignment between needs and services provided. We hypothesized that women who received higher quality counseling at baseline would be more likely to share their informational needs were met to a greater degree at follow-up.

Finally, our second person-centered measure comprised a binary indicator of care-seeking for the management of contraceptive side effects. Among women who reported experiencing side effects, we asked, “After experiencing these side effects, did you talk with a health care provider about them?” (yes/no). We used this measure given our hypothesis that women who received more comprehensive, client-centered, and supportive information during counseling would be more likely to seek care, if they experienced side effects, and would also be more likely to return to a healthcare provider in the future.

We also measured several potential confounders based on their theoretical and empirical significance in women’s contraceptive counseling experiences, including sociodemographic and visit characteristics (Walker et al. 2021; Mickler et al. 2021; Schwandt, Speizer, and Corroon 2017; Calhoun et al. 2013; Dieci et al. 2021). Sociodemographic characteristics included age, educational attainment, marital/union status, and parity. Visit characteristics included reason for facility visit, outcome of service, receipt of desired method, and method type received (among users), as well as contraceptive use status immediately before the initial family planning visit, which was measured using three categories that distinguished women who were previously (1) using the same method type as the one received on the day of interview, (2) using a different method type as the one received on the day of interview, and (3) were not using any method. We also assessed the type of method received or prescribed, including long-acting methods (IUDs, implants, sterilization) versus short-acting methods (injectables, pills, emergency contraception, condoms, traditional methods).

### Analytic Samples

We used three analytic samples to explore our study aims based on the full sample of women who participated in the baseline survey. Our first analytic sample for cross-sectional analysis—used to examine validity and reliability of the QCC-10—consisted of all women who completed the baseline survey following their facility visit and receipt of information on family planning, regardless of the outcome of visit (e.g., left the facility with a method, prescription, or no form of contraception); altogether, this included 7,105 women (Burkina Faso: *n* = 960; Kenya: *n* = 4,841; Kano: *n* = 746; and Lagos: *n* = 558). A wide range in sample sizes is primarily due to different numbers of enumeration areas for the population-based household surveys, around which the facility surveys and client exit interviews are designed (Zimmerman et al. 2017).

Our second analytic sample for longitudinal analysis—used to assess the predictive utility of the QCC-10 for continued protection from unintended pregnancy and informational needs about contraception being met at follow-up—included women who received a contraceptive method or prescription at baseline and completed the follow-up interview; this totaled 4,692 women (Burkina Faso: *n* = 597; Kenya: *n* = 3,270; Kano: *n* = 479; and Lagos: *n* = 346). Our third analytic sample for longitudinal analysis—used to explore the association between baseline QCC-10 scores and care-seeking for contraceptive side effects—was restricted to women who received a contraceptive method or prescription at baseline, completed the follow-up interview, and reported experiencing side effects with their baseline method, including 2,057 women (Burkina Faso: *n* = 244; Kenya: *n* = 1,375; Kano: *n* = 246; and Lagos: *n* = 192). Samples were limited to women with complete data for outcome measures, which accounted for approximately 96 percent of those eligible in each geography.

### Analysis

We first used cross-sectional data from baseline surveys to explore the validity and reliability of the QCC-10 scale in each of the four sub-Saharan African geographies. We applied classical test theory, following psychometric approaches outlined by DeVellis and Netemeyer et al., to assess item properties and determine if the QCC-10’s original scale properties translated to new geographies (DeVellis 2016; Netemeyer, Bearden, and Sharma 2003). We calculated descriptive statistics to summarize responses for each item (e.g., category frequencies, proportions, means, standard deviations) and composite scale scores (simple total divided by the total number of items), separately for each geography.

Next, we used exploratory factor analysis (EFA) to examine construct validity and examined Cronbach’s alpha values to assess internal consistency reliability. EFA included use of polychoric correlation matrices from principal components analysis (PCA) in each geography to identify the number of factors. Application of polychoric PCA estimated the polychoric and polyserial correlations among the QCC-10 items to perform EFA on the resulting correlation matrix. This approach offered a robust alternative to ordinary EFA for circumstances in which item responses violate assumptions of normality, such as those for QCC-10 items and related quality measures. Factor structures and dimensionality of the QCC-10 were assessed through eigenvalues (>1.0) and Scree plots (the “elbow” distinction in the plot of eigenvalues indicating the number of factors to retain). We examined the strength of each item via factor loadings. Items with low (*λ* < 0.40) or very high (*λ* > 0.90) factor loadings were flagged for potential exclusion due to poor fit and item redundancy, respectively; Cronbach’s alpha values *α* > 0.6 were considered acceptable for evidence of internal consistency reliability (DeVellis 2016; Netemeyer, Bearden, and Sharma 2003). We examined convergent validity—or how scores on the scale correlated with other outcomes theorized to capture a similar construct—by evaluating associations between QCC-10 scores and highest satisfaction ratings. Associations were assessed using bivariable and multivariable logistic regression models with robust standard errors to account for clustering of women within facilities.

Next, we used longitudinal data from phone-based follow-up surveys in each of the four sub-Saharan African geographies to assess the predictive utility of the QCC-10 for understanding reproductive health experiences in the first six months after the baseline family planning visit. Among women who received a contraceptive method or prescription for a method at baseline and completed the follow-up interview, we first examined the distribution of women’s reproductive outcomes and contraceptive use statuses at follow-up. Next, we used bivariable and multivariable logistic regression models to assess the extent to which baseline QCC-10 scores predicted women’s continued protection from unintended pregnancy, the extent that their informational needs were met at follow-up, and, among women who reported experiencing side effects with their baseline contraceptive method, whether they sought care from a healthcare provider to manage their side effects. Longitudinal regression analyses adjusted for loss-to-follow-up between baseline and follow-up, propensity of phone ownership, and clustering of women within facilities; similar weighting approaches have been applied in other longitudinal analyses using PMA data (Karp et al. 2021; Zimmerman et al. 2022).

## RESULTS

### Participant and Visit Characteristics

Table 2 presents participant and visit characteristics of women in the baseline sample. Approximately half of women across geographies were aged 25–34 years, and most were married or in-union (ranging from 84.4 percent in Kenya to 99.3 percent in Kano, Nigeria). Educational attainment varied widely across geographies, with 38.8 percent of women completing no education in Burkina Faso compared to 41.4 percent completing higher than secondary in Lagos. Most women had at least one child, with 55.7 percent of women in Kano having four or more children. Nearly all women reported family planning as the primary reason they visited the facility (84.9 percent in Kenya to 97.3 percent in Kano) and most left the facility with a contraceptive method (88.4 percent in Kenya and Lagos to 91.6 percent in Kano), though this was considerably lower in Burkina Faso, where 60.8 percent received a method and one-quarter received method prescriptions (26.2 percent). The majority of women who were visiting the facility for non-follow-up care (i.e. a new method) received their desired method (90.7 percent in Kenya to 99.4 percent in Kano). Across geographies, injectables were the most common method type received or prescribed, constituting roughly half of the women in each site, followed by implants (20.4 percent in Burkina Faso to 29.9 percent in Lagos).

### Psychometric Properties

Table 3 shows distributions, means, standard deviations, and factor loadings of QCC-10 items and internal consistency reliability of the QCC-10 scale by geography. The proportion of women strongly endorsing each item varied widely across geographies and by quality domain. Overall, women in Lagos reported the highest level of agreement with each of the statements relating to the *information exchange* and *interpersonal relations* domains (ranging from 40.8 percent for the “explain” item about how to use methods to 57.0 percent for the “express_self” item about encouragement to ask questions and express concerns), while agreement was consistently lowest among women in Burkina Faso (ranging from 19.9 percent for the “body_react” item, reflecting counseling on side effects, to 40.0 percent to the “no interrupt” item about ensuring visit privacy). The only exception of this pattern was for the “body_react” item, which was strongly endorsed by even fewer women in Kano (17.9 percent). Two items reflecting the domain of *disrespect and abuse* were most frequently endorsed by women in Kano (5.5 percent completely agreeing with the “prov_insist” item, that they felt pressured to use the provider’s preferred method, and 4.0 percent reporting they felt scolded because of their marital status), followed by women in Kenya (2.0 percent and 1.2 percent completely agreeing, respectively). Item means ranged from 2.9 to 3.5 (out of 4) in Burkina Faso, 3.2–3.4 in Kenya, 3.1–3.4 in Kano, and 3.4–3.6 in Lagos. Overall QCC-10 scores ranged from 3.1 (out of 4) in Burkina Faso to 3.5 in Lagos, with higher scores indicating greater quality counseling.

EFA using polychoric PCA correlation matrices indicated a two-factor solution across the four contexts, with two eigenvalues greater than one (Table 3). Scree plots in each geography illustrated an “elbow” at two, providing further evidence of a two-factor solution. In applying the two-factor solution in each geography, all but two items representing the *disrespect and abuse* domain (items: prov_insist, scold_marital) loaded within an acceptable range (*λ*: 0.4–0.9) onto Factor 1, while the *disrespect and abuse* items loaded strongly onto Factor 2 (*λ*: 0.7–0.9). Internal consistency reliability, measured via Cronbach’s alpha, was moderate-to-high across contexts for the composite scale combining items from both factors, ranging from *α* = 0.73 in Burkina Faso to *α* = 0.91 in Lagos. We conducted sensitivity analyses, first removing each of the two *disrespect and abuse* items, to determine if reliability improved; Cronbach’s alpha did not substantially change with the modified scale. We also assessed subscale reliability for each factor. Alphas for Factor 1 (*information exchange* and *interpersonal relations* items only) ranged from 0.82 in Kano, Nigeria to 0.93 in Kenya, while those for Factor 2 (*disrespect and abuse* items only) ranged from 0.58 in Burkina Faso to 0.86 in Lagos, Nigeria (data not shown). As with the original QCC-10 short scale development, and given high alphas for the 10-item measure, we retained the two *disrespect and abuse* items within the composite measure. This approach captured aspects of negative counseling experiences to preserve content validity of the QCC-10 scale and alignment with the measurement framework (Holt, Karp, et al. 2021; Holt et al. 2019).

### Convergent Validity

Table 4 presents findings from the convergent validity analysis examining the association between QCC-10 scores and satisfaction ratings. The proportion of women reporting highest satisfaction with their family planning visit ranged from 40.1 percent of women in Burkina Faso to 69.6 percent in Kano, Nigeria. In all four geographies, women’s QCC-10 scores were highly correlated with self-reported high satisfaction; unadjusted odds ratios (ORs) ranged from 3.52 in Lagos, Nigeria to 21.14 in Kenya (all *p* < 0.001). Multivariable models, adjusting for women’s sociodemographic characteristics, confirmed results of bivariate analysis; adjusted ORs ranged from 3.51 (95 percent CI: 1.90–6.45) in Lagos to 21.20 (95 percent CI: 13.62–32.99) in Kenya. Each one-unit increase in a woman’s QCC-10 mean score (out of the possible range of 0–4) was associated with higher odds of her reporting that she was “very satisfied” with her family planning visit.

### Reproductive and Person-Centered Outcomes at Follow-Up

Table 5 shows descriptive results related to women’s reproductive outcomes at follow-up. Contraceptive continuation six months after the initial contraceptive visit was high, with approximately 80 percent of women in each geography using their baseline method at follow-up. After accounting for women’s reasons for nonuse and discontinuation, the proportion of women who had continued protection from unintended pregnancy rose to 91.9 percent in Kano, and 96.0 percent in Kenya. Roughly half of women experienced side effects related to their contraceptive method. Among them, the proportion that talked to a health provider about their side effects ranged from 47.5 percent in Burkina Faso to 73.1 percent in Kano.

**FIGURE 1:**
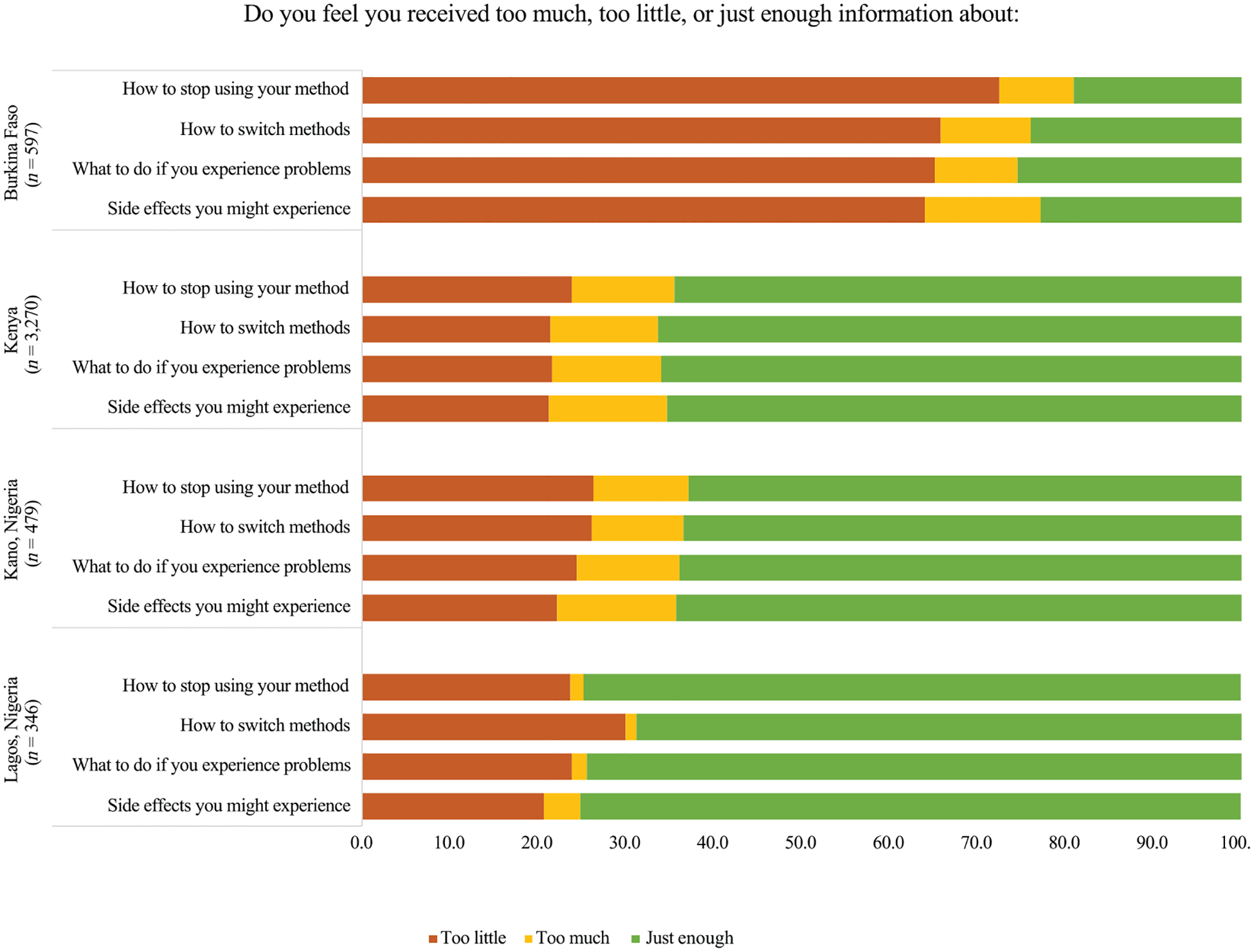
Distributions of women’s contraceptive informational needs being met at follow-up by counseling component and geography

Figure 1 illustrates distributions of women’s information needs being met at follow-up by counseling component and geography. The fulfillment of women’s contraceptive informational needs six months after care varied widely across study contexts, with greater informational needs being met in Kenya, and Kano and Lagos, Nigeria. In contrast, most women in Burkina Faso consistently reported receiving “too little” information about each of the counseling components, ranging from 64.0 percent for potential side effects to 72.5 percent for how to stop using your method. On average, women’s scores for their information needs being met at follow-up ranged from 2.24 (out of 8) in Burkina Faso to 5.89 in Lagos, Nigeria (Table 5).

Table 6 presents multivariable regression results from separate regression models predicting the adjusted associations between women’s baseline QCC-10 scores and reproductive health measures at follow-up. Quality of contraceptive counseling at baseline was not associated with women’s continued protection from unintended pregnancy at follow-up in any geography. In contrast, we observed a consistent, positive relationship between women’s QCC-10 mean scores at baseline with their reported informational needs being met at follow-up in Burkina Faso and Kenya. Each one-unit increase in a woman’s QCC-10 mean score was associated with a one-point increase in her informational needs met score in Burkina Faso (a*β* = 1.06, 95 percent CI: 0.63–1.49) and Kenya (a*β* = 1.02, 95 percent CI: 0.79–1.25). The reverse was true in Kano, Nigeria, where results indicated a negative association between higher quality counseling and informational needs being met (a*β* = −0.94, 95 percent CI: −1.82 to −0.04); similar relationships were observed in Lagos, Nigeria, though results were not statistically significant. Care-seeking for contraceptive side effects had mixed associations across geographies. Women’s likelihood of talking with a provider about their side effects did not differ by baseline QCC-10 scores for those in Burkina Faso, and Kano and Lagos, Nigeria. In Kenya, however, women’s odds of seeking care for experienced side effects increased 42 percent with each one-unit increase in a woman’s QCC-10 mean score (aOR = 1.42, 95 percent CI: 1.02, 1.98). Sensitivity analyses, examining associations between the two *disrespect and abuse* items separately from the eight *informational exchange* and *interpersonal relations* items, produced similar results, including mixed findings across geographies (Online Appendix Table T2).

## DISCUSSION

Measurement of quality in family planning services has long been a focus of the reproductive health field. Despite the recent development of multiple metrics for exploring technical or structural dimensions of quality, less emphasis has been placed on understanding quality of contraceptive counseling using validated tools, particularly those that capture negative aspects of care. Measures that assess client perspectives of contraceptive services offer critical insight for quantifying and disentangling how person-centered care is being delivered to protect human rights in reproductive healthcare.

The Quality of Contraceptive Counseling short scale (QCC-10) measures client experiences of contraceptive counseling across diverse contexts by adopting a human rights perspective, with direct measurement of negative experiences and centering explicitly on client preferences, values, and needs in the evaluation of family planning counseling. This person-centered measure adds to a growing field of research on quality in contraceptive care by offering a shorter, 10-item tool for inclusion in routine monitoring and evaluation or within a larger survey of contraceptive users.

Using data from family planning clients in Burkina Faso, Kenya, and two states in Nigeria, we find that the QCC-10 scale is a psychometrically robust measure for examining quality in contraceptive services across the domains of *information exchange*, *interpersonal relations*, and *disrespect and abuse*. Results also illustrate that the QCC-10 holds predictive utility for understanding aspects of person-centered measures of reproductive health over time in certain contexts, yet mixed findings underscore the need for future work in this area. Efforts to improve understanding of how the QCC-10 scale relates to person-centered measures of reproductive health may be strengthened by inclusion of a more robust suite of measures that reflect women’s self-defined contraceptive needs than those available in our study.

Our findings contribute to a growing body of evidence about the linkages between high-quality contraceptive care and positive reproductive health outcomes. We adopted a person-centered framework for our investigation, exploring outcomes that reflected the goals of human rights-based care in family planning services. By measuring whether women’s informational needs were met and, among those experiencing side effects, if they sought care to manage those experiences, we aimed to align our evaluation of quality more closely with individuals’ reproductive preferences, values, and needs. We echo recommendations of other scholars in applying this approach and seek to draw the field away from programmatic-driven outcomes and toward more person-centered measures (Senderowicz 2020; Holt et al. 2020). Historically, researchers have relied on programmatic measures, like use of highly effective methods or contraceptive continuation, to explore the impact of receiving high-quality care (Chakraborty et al. 2019; Jain, Aruldas, Tobey, et al. 2019; Jain, Aruldas, Mozumdar, et al. 2019; Dey et al. 2021), yet women may choose to stop using contraception for a variety of reasons that still align with their reproductive goals and preferences. We posited that women’s reflections on their counseling experiences, months after their initial visit, and their care-seeking practices related to the management of side effects would offer better insights into the impact of higher quality contraceptive counseling than programmatic measures.

We found that the vast majority of women continued contraceptive use or discontinued for reasons indicating they were not at risk of unintended pregnancy at follow-up. Findings suggest that contraceptive services in study locations largely protect contraceptive users from unintended pregnancy, though we lacked direct measures of users’ self-defined assessment of whether their needs were met. We found no association between receiving higher quality counseling at baseline and being at risk of unintended pregnancy at follow-up. Instead, we observed positive and significant associations between higher QCC-10 scores and women reporting their informational needs were met approximately six months after their visit to Burkina Faso and Kenya, but the reverse association in Kano, Nigeria. In Lagos, Nigeria, the lack of relationship between QCC-10 scores and reproductive outcomes may be partially attributable to the high degree of quality reported across all QCC-10 items and the large number of women who reported receiving “just enough” information for meeting their contraceptive informational needs at follow-up. In Kano, this association may reflect challenges in implementing the QCC-10 in this context, both where contraceptive use and health care utilization are lower than other settings and where the psychometric properties of the scale were weakest. Despite these challenges, overall, these findings suggest that the QCC-10 scale may be useful as a tool for identifying opportunities for quality improvement to enhance delivery of comprehensive and person-centered services.

Contraceptive side effects are experienced by a large proportion of contraceptive users, including roughly half of women in our sample. The management of these experiences—or lack thereof—has been cited as one of the primary reasons women discontinue their methods. We anticipated that, among women who experienced side effects, those who received higher quality contraceptive counseling would be more likely to seek help from a health provider. While we found that this was true for women in Kenya, null findings in Burkina Faso and two states in Nigeria underscore the complexity of this relationship. Anecdotal reports in Nigeria suggest that when women have a provider who they perceive as delivering high-quality, friendly, and supportive care, they are more likely to seek care at the same facility when they experience side effects.

A few explanations emerge for why quality of counseling may not clearly relate to contraceptive care-seeking among women in our study. First, women who received higher quality care may be more likely to self-manage their side effects without consulting a health provider. Key to comprehensive counseling is the discussion of potential side effects and what to do if they are experienced. It is possible that women receiving higher quality counseling were more likely to anticipate side effects before they occurred and manage or tolerate them without provider engagement. A second explanation lies in the potential of social networks. Regardless of their contraceptive counseling experiences, women may rely more heavily on their friends, peers, and family members, to source recommendations for mitigating side effects, rather than reaccessing health services due to convenience, trust, and value placed on experiences of close confidantes. One study in Madagascar found that women with social networks of individuals who they consulted about contraception were more likely to be using a method (Comfort et al. 2021). And, finally, the context of facilities and their accessibility, which was not explored in this paper, may have played an important role in women’s decision-making about whether to return to a facility for follow-up care. For example, clients receiving care from higher volume facilities may experience longer waiting times, pay higher out-of-pocket fees, and receive shorter visits with providers, potentially discouraging them from returning to a provider for follow-up care. Each of these potential explanations warrants further investigation, particularly through research that leverages data from facilities and women, to disentangle the relationship between high-quality contraceptive counseling, enabling service delivery environments, and women’s contraceptive care-seeking practices.

We also highlight measurement challenges and offer considerations related to the development of psychometrically robust metrics of quality in family planning services. In our study, two items reflecting biased or directive counseling showed distinct psychometric results, particularly in Kano, Nigeria where these negative experiences were most frequently reported. Despite our hypotheses of the QCC-10 scale’s unidimensionality, we found evidence of two factors—isolating the *disrespect and abuse* items as a separate factor—in multiple geographies. We posit that this finding may have been attributable, in part, to the low proportion of women reporting provider coercion or biased counseling in our study samples, which may have affected the psychometric properties of items. We also recognize that our sample was comprised of predominantly married women, thus provider bias that has been documented against young, unmarried women in these, and other, contexts may have been insufficiently captured in our sample and inadequately reflected in our results. As with the original scale reduction and following psychometric assessment, we decided to retain the two *disrespect and abuse* items in our exploration of the composite QCC-10 scale, thereby preserving the content validity and alignment of the scale with the measurement framework, which explicitly acknowledges the role of adverse experiences in the experience of contraceptive care (Holt, Dehlendorf, and Langer 2017; Holt et al. 2019). These psychometric challenges underline a key tension in the measurement of quality care to ensure indicators do not ignore negative experiences that impede the delivery of person-centered services (Reichenbach, Hardee, and Harris 2016; Senderowicz 2019; Tumlinson, Okigbo, and Speizer 2015; Solo 2019). Additionally, we conducted sensitivity analyses and found minimal improvement in the scale with the removal of the two *disrespect and abuse* items, providing further support for their retention. Together, these findings underscore the complexity of measure development for quality of contraceptive care and highlight opportunities for future research that explores these issues in other geographies.

### Limitations and Strengths

This study is not without limitations. We implemented phone-based follow-up interviews of women approximately six months after their initial family planning visit. This approach may limit the relevance of our predictive validity findings for populations without access to a phone, which accounted for nearly one-quarter of eligible women in Kano, Nigeria. We used survey weights, adjusting for a woman’s propensity for phone ownership, to address some of this variation in our final sample, though we acknowledge that women who lack access to phones may experience contraceptive care and use differently than women we surveyed. Additionally, data used in this analysis come from PMA’s client exit interview survey, which is conducted in facilities considered “medium-to-high volume”, serving three or more family planning clients per day. Results do not reflect the experiences of women in study geographies who receive care from smaller facilities where quality of contraceptive care may differ considerably from larger, higher volume facilities.

Finally, efforts to understand the implications of quality in contraceptive care should be grounded in a suite of person-centered outcomes to reflect women’s self-identified contraceptive needs and the ways in which services support them in achieving their reproductive goals. While we sought to measure aspects of person-centeredness in our longitudinal measures, we lacked direct questions about if women felt their contraceptive needs were met at follow-up. Unlike our measures capturing informational needs and management of side effects at follow-up, our proxy of an individual’s continued protection from unintended pregnancy reflected our judgment, as researchers, about women’s reasons for nonuse and assumed that all women seeking contraceptive services at baseline wanted to avoid pregnancy in the near term. A lack of explicit measurement about women’s pregnancy and contraceptive preferences limited the person-centeredness of this measure. Future research should leverage innovative, person-centered measures to better understand the impact of high-quality counseling on women’s ability to achieve their reproductive goals.

Despite these limitations, our study makes an important contribution to the reproductive health field. We investigated a new, 10-item measure for examining quality and person-centeredness of contraceptive services in four diverse geographies across three sub-Saharan African countries, enhancing the robustness of our validation and potential utility of the tool in these, and similar, geographies. We also used rich, longitudinal data and rigorous psychometric methods to evaluate the new scale and understand the potential impact of high-quality contraceptive counseling on women’s contraceptive needs over time. Finally, our study examined person-centered measures of reproductive health to situate the quality of family planning interactions within the context of individual’s reproductive lives. In addition to its utility for research on quality in contraceptive care, the QCC-10 can serve as a tool for programmatic, monitoring, and evaluation efforts that seek to enhance the delivery of person-centered contraceptive care. We used a simple analytic technique for the QCC-10 score—an average of individual item scores—that could be easily applied for use within health facilities and family planning programs. Individual item scores can also be examined to identify specific opportunities for intervention with providers and health services more broadly.

## CONCLUSION

This study validates the QCC-10 scale, a novel 10-item measure of quality and person-centeredness in family planning services. Psychometric evaluation of the scale indicates the QCC-10 is a robust measure for the assessment of key quality domains, including *information exchange*, *interpersonal relations*, and *disrespect and abuse*, in family planning services, though some inconsistencies in Nigeria warrant further investigation. Overall, the QCC-10 offers a promising new approach for documenting understudied dimensions of quality in contraceptive counseling in sub-Saharan Africa and is useful in examining person-centered reproductive health outcomes over time. Further testing is needed to validate the measure in new contexts, particularly for understanding important dimensions of *disrespect and abuse*, and examine potential social inequities in the experience of person-centered contraceptive care. Researchers and programs can integrate the QCC-10 into surveys across the diverse geographies studied to measure and improve the quality of reproductive health services to ensure they align with the contraceptive needs and preferences of individuals seeking care.

## Supplementary Material

Supplementary Tables

## Figures and Tables

**TABLE 1:** Variable names and item wording for Quality of Contraceptive Counseling (QCC-10) scale items by domain

Variable name	Item wording
*Information exchange (five items)*	
personal	The provider asked me questions to provide counseling that fit me personally
info	I received all the information I wanted to know about my options for contraception
body_react	I understand how my body might react to using contraception
explain	I understand how to use method(s) we talked about during the consultation
opinion	I was able to give my opinion about what I needed
*Interpersonal relations (three items)*	
express_self	I felt encouraged to ask questions and express my concerns
no_interrupt	Provider tried to ensure there were no interruptions during our session
enough_time	The provider gave me the time to consider the contraceptive options
*Disrespect and abuse (two items)*	
prov_insist	I felt pressured by the provider to use the method they wanted me to use^a^
scold_marital	I felt scolded because of my marital status^a^

RESPONSE OPTIONS: “Completely agree” (coded as 4); “agree” (coded as 3); “disagree” (coded as 2); “completely disagree” (coded as 1). Response options for the two disrespect and abuse items and exact item wording for several items varied slightly from the original QCC-10 paper (Holt et al., 2022), given adjustments made by the Performance Monitoring for Action (PMA) research teams to accommodate in-country preferences.

a Items reverse scored with “completely agree” coded as 1 and “completely disagree” coded as 4; higher scores indicated higher quality counseling.

**TABLE 2:** Baseline sociodemographic and visit characteristics by geography

	Burkina Faso (*n* = 960)		Kenya (*n* = 4,841)		Kano, Nigeria (*n* = 746)		Lagos, Nigeria (*n* = 558)	
	*n*	%	*n*	%	*n*	%	*n*	%
*Sociodemographic characteristics*								
Age								
15–24	337	35.1	1,483	30.6	191	25.7	37	6.6
25–34	430	44.8	2,400	49.6	340	45.5	262	47.0
35–49	193	20.1	958	19.8	215	28.8	259	46.4
Parity								
0–1^a^	327	34.1	1,435	29.7	102	13.6	68	12.1
2–3	381	39.7	2,214	45.8	228	30.7	310	55.5
4+	252	26.3	1,190	24.6	416	55.7	180	32.2
Education								
Never	372	38.8	99	2.1	124	16.6	10	1.8
Primary	218	22.7	2,036	42.1	117	15.8	47	8.4
Secondary	331	34.5	1,771	36.6	379	50.7	270	48.4
Higher	39	4.1	935	19.3	125	16.8	231	41.4
Marital status								
Not in-union	137	14.1	755	15.6	5	0.7	13	2.3
In union	837	85.9	4,091	84.4	741	99.3	545	97.7
*Visit characteristics*								
Family planning reported as the main reason for a facility visit								
Yes	900	93.8	4,111	84.9	727	97.3	509	90.9
No	60	6.3	730	15.1	19	2.6	51	9.1
Outcome of service								
Method	584	60.8	4,277	88.4	684	91.6	495	88.4
Prescription	251	26.2	256	5.5	19	2.5	3	0.5
Neither	125	13.0	298	6.2	44	5.9	62	11.1
Received desired method^b^								
Yes	815	94.8	4,159	90.7	692	99.4	453	98.1
No	45	5.2	429	9.4	4	0.6	9	1.9
Method type received or prescribed^c^								
Female sterilization			4	0.1	1	0.1		
Implant	170	20.4	1,277	28.1	197	28.0	149	29.9
IUD	38	4.6	174	3.8	33	4.7	28	5.6
Injectables	505	60.5	2,175	47.9	394	56.1	242	48.6
Pills	121	14.5	802	17.7	76	10.8	67	13.5
Emergency			16	0.4				
contraception								
Male condom			71	1.6	1	0.1	11	2.2
Female condom			6	0.1			1	0.2
Diaphragm					1	0.1		
Standard days/Cycle			3	0.1				
beads								
Lactational			7	0.2				
amenorrhea method								
Rhythm method			1	<0.1				
Withdrawal	1	0.1	2	<0.1				
Other traditional			4	0.1				
methods								

a Parity category 0–1 pooled due to a small number of nulliparous women in the sample.

b Among women who were not accessing services for a follow-up visit.

c Among women who received a method or prescription for a method.

**TABLE 3:** Item distributions, means, standard deviations, and factor loadings and scale internal consistency reliability by geography

	Burkina Faso (*n* = 960)	Kenya (*n* = 4,841)	Kano, Nigeria (*n* = 746)	Lagos, Nigeria (*n* = 558)
	Agree^a^ (%)	Disagree^b^ (%)	Agree^a^ (%)	Disagree^b^ (%)	Agree^a^ (%)	Disagree^b^ (%)	Agree^a^ (%)	Disagree^b^ (%)
**Information exchange**								
personal	23.3	3.6	35.7	0.9	31.7	0.1	48.0	0.0
info	26.8	3.3	34.4	0.7	36.6	0.1	49.1	0.0
body_react	19.9	4.4	32.7	0.9	17.9	0.1	44.4	0.2
explain	21.5	2.3	34.0	0.6	24.2	0.1	40.8	1.3
opinion	28.5	2.1	33.8	0.4	23.2	0.3	43.4	1.4
**Interpersonal relations**								
express_self	27.4	4.6	41.6	0.3	42.0	0.3	57.0	0.0
no_interrupt	40.0	2.0	41.4	0.3	34.1	0.1	48.8	0.2
enough_time	24.5	3.0	35.8	0.3	28.5	0.4	48.1	0.4
**Disrespect and abuse**								
prov_insist^c^	2.2	42.0	2.0	35.2	5.5	28.8	0.5	64.0
scold_marital^c^	0.4	53.7	1.2	37.3	4.0	28.1	0.5	63.5
	Mean (SD)	Load^d^	Mean (SD)	Load^d^	Mean (SD)	Load^d^	Mean (SD)	Load^d^
**Information exchange**								
personal	2.93 (0.78)	0.79	3.29 (0.60)	0.83	3.29 (0.51)	0.78	3.46 (0.54)	0.85
info	3.01 (0.77)	0.86	3.27 (0.60)	0.89	3.35 (0.50)	0.80	3.47 (0.53)	0.92
body_react	2.87 (0.77)	0.75	3.24 (0.61)	0.85	3.15 (0.43)	0.79	3.42 (0.55)	0.88
explain	3.01 (0.68)	0.78	3.29 (0.55)	0.89	3.23 (0.45)	0.76	3.40 (0.51)	0.84
opinion	3.09 (0.72)	0.54	3.29 (0.56)	0.82	3.21 (0.47)	0.63	3.42 (0.52)	0.80
**Interpersonal relations**								
express_self	2.95 (0.83)	0.71	3.37 (0.58)	0.82	3.38 (0.56)	0.57	3.56 (0.52)	0.83
no_interrupt	3.28 (0.69)	0.58	3.39 (0.55)	0.76	3.32 (0.52)	0.70	3.47 (0.54)	0.86
enough_time	2.93 (0.78)	0.81	3.31 (0.57)	0.89	3.25 (0.52)	0.71	3.45 (0.57)	0.85
**Disrespect and abuse**								
prov_insist^b^	3.33 (0.67)	0.75	3.28 (0.61)	0.88	3.14 (0.72)	0.72	3.62 (0.53)	0.91
scold_marital^b^	3.51 (0.56)	0.68	3.33 (0.57)	0.65	3.15 (0.69)	0.70	3.62 (0.52)	0.83
**Eigenvalue for Factor 1**		**4.43**		**6.05**		**4.28**		**6.95**
**Eigenvalue for Factor 2**		**0.97**		**1.10**		**1.50**		**0.86**
**Composite QCC-10 Mean**		**3.08 (0.46)**		**3.31 (0.42)**		**3.25 (0.30)**		**3.48 (0.42)**
**Composite QCC-10 Alpha**		**0.83**		**0.89**		**0.73**		**0.91**

a Load = factor loading of each item onto Factor 1, except for disrespect and abuse items, which are indicated for Factor 2.

b Items reverse scored; higher scores indicate higher quality counseling. Mean scores range: 1–4. SD = standard deviation.

c Agree = completely agree.

d Disagree = completely disagree.

**TABLE 4:** Convergent validity by geography: Frequency, percent, and odds of reporting highest satisfaction rating for family planning visit, predicted by Quality of Contraceptive Counseling (QCC-10) scale, by geography

	Burkina Faso (*n* = 960)			Kenya (*n* = 4,841)			Kano, Nigeria^a^ (*n* = 746)			Lagos, Nigeria^a^ (*n* = 558)		
	*n* (%)	OR (*p*-value)	aOR (95% CI)	*n* (%)	OR (*p*-value)	aOR (95% CI)	*n* (%)	OR (*p*-value)	aOR (95% CI)	*n* (%)	OR (*p*-value)	aOR (95% CI)
Highest satisfaction rating for initial family planning visit	385 (40.1)	6.37 (*p* < 0.001)	7.47*** (5.10–11.0)	2,334 (48.2)	21.14 (*p* < 0.001)	21.20*** (13.62–32.99)	520 (69.6)	7.44 (*p* < 0.001)	8.00*** (3.06–20.89)	384 (68.6)	3.52 (*p* < o.ooi)	3.51*** (1.90–6.45)

NOTE: OR = odds ratio; aOR = adjusted odds ratio, adjusted for age, parity, marital status (^a^except in Nigeria where nearly all women were in-union), and education. Robust confidence interval in parentheses, adjusting for clustering of women within facilities.

*** *p* < 0.01,

** *p* < 0.05,

* *p* < 0.1.

Key predictor: QCC-10 scale score.

**TABLE 5:** Among women who completed follow-up interviews, women’s reproductive outcomes and behaviors at follow-up

	Burkina Faso (*n* = 597)	Kenya (*n* = 3,270)	Kano, Nigeria (*n* = 479)	Lagos, Nigeria (*n* = 346)
**Reproductive outcomes, *n***				
1. Unintended pregnancy. Women who:				
experienced method failure, currently pregnant	1	21	1	2
discontinued due to perceived low risk, currently pregnant	0	2	0	0
2. Discontinued while in need. Women who:				
discontinued but did not mention reasons indicating no need	30	107	35	19
3. Discontinued due to no need. Women who:				
were currently pregnant, intended	2	22	10	0
were currently pregnant, without citing reasons indicating lack of need	2	6	8	1
discontinued due to desire to become pregnant	10	29	5	6
discontinued due to perceived low risk of pregnancy^a^	4	37	2	3
4. Switched methods. Women who:				
started using the baseline, discontinued, using a method currently	34	332	28	32
did not start using the baseline method provided, but were using a method at baseline, and are using a method currently^b^	26	59	7	8
5. Women who adopted a new method^b^	6	10	2	2
6. Women who continued using their baseline method	486	2647	361	275
**Baseline contraceptive method use, *n* (%)**				
Not using method	115 (19.3)	624 (19.1)	105 (21.9)	73 (21.1)
Still using method	482 (80.7)	2,646 (80.9)	374 (78.1)	273 (78.9)
**Continued protection from unintended pregnancy, *n* (%)**				
Not protected	31 (5.2)	130 (4.0)	39 (8.1)	21 (6.1)
Protected	566 (94.8)	3,140 (96.0)	440 (91.9)	325 (93.9)
**Informational needs met score, (range: 0–8), mean (SD)**	2.24 (2.53)	5.74 (2.76)	5.55 (2.91)	5.89 (2.55)
**Care-seeking for contraceptive side effects, *n* (%)**				
Did not seek care	313 (52.5)	1,527 (46.7)	129 (26.9)	137 (39.6)
Sought care	284 (47.5)	1,743 (53.3)	350 (73.1)	209 (60.4)

a “Infrequent sex/husband/partner away” or “Difficult to get pregnant/menopausal”.

b Did not start using the baseline method provided at their counseling visit.

**TABLE 6:** Among women who received a prescription or method at baseline, adjusted associations between women’s baseline Quality of Contraceptive Counseling (QCC-10) scale scores and reproductive outcomes at follow-up, by geography—results from separate multivariable regression models

	Burkina Faso (*n* = 597)	Kenya (*n* = 3,270)	Kano, Nigeria (*n* = 479)	Lagos, Nigeria (*n* = 346)
**Model and reproductive outcome**				
**Model 1: Continued protection from unintended pregnancy**	0.38	1.04	2.23	1.14
aOR (95% CI)	(0.61–3.12)	(0.68–1.60)	(0.62–7.99)	(0.36–3.57)
**Model 2: Informational needs met**	1.06***	1.02***	−0.94	−0.14
a*β* (95% CI)	(0.63–1.49)	(0.79–1.25)	(−1.82 to −0.04)	(−1.15–0.86)
**Model 3: Care-seeking for side effects** ^a^	1.14	1.42**	2.53	0.81
aOR (95% CI)	(0.67–1.94)	(1.02–1.98)	(0.88–7.29)	(0.37–1.77)

NOTE: Models adjusted for age, educational attainment, marital status, and prior contraceptive method use. Marital status is not included in Nigeria, due to small variation. Ref=reference group. aOR=adjusted odds ratio. a*β*=adjusted linear regression coefficient.

a Analyses restricted to women who with any side effects of their baseline method.

*** *p* < 0.01,

** *p* < 0.05,

* *p* < 0.1.

Estimates adjusted for lost-to-follow-up analytical weights.

## Data Availability

The data that support the findings of this study are openly available via www.pmadata.org.
