# Supplementary material for: Validation and Predictive Utility of a Person-Centered Quality of Contraceptive Counseling (QCC-10) Scale in Sub-Saharan Africa: A Multicountry Study of Family Planning Clients and a New Indicator for Measuring High-Quality, Rights-Based Care
Source: Stud Fam Plann. Author manuscript; Available in PMC 2024 Jun 5. (PMC11152181; doi:10.1111/sifp.12229)
Supplement: Supplementary Tables [file NIHMS1991485-supplement-Supplementary_Tables.pdf]

APPENDIX TABLE T1 Number of baseline clients, eligible clients for follow-up, and clients who completed phone follow-up interview

	Burkina Faso	Kenya	Kano, Nigeria	Lagos, Nigeria
Number of clients at baseline	911	4115	695	509
% of baseline clients who received:				
Contraceptive methods	61.8	89.7	94.2	89.2
Prescription for contraceptive methods	25.8	5.0	1.2	0.0
Neither	12.4	5.3	4.6	10.8
Eligible for follow up	87.6	94.7	95.4	89.2
Number of clients eligible for phone follow up	798	3898	663	454
% of eligible clients who:				
Were willing to participate in the follow up	97.2	98.4	93.5	96.3
+ Had access to a phone	87.6	94.1	77.2	90.5
+ Were contacted successfully	78.3	85.1	72.2	78.4
+ Consented for the interview	77.6	85.1	72.2	77.5
+ Completed the interview	77.6	85.0	72.2	77.3
**Number of clients who completed phone follow-up interview**	**619**	**3,313**	**481**	**351**

APPENDIX FIGURE A1 Follow-up interview questions to determine outcome variables

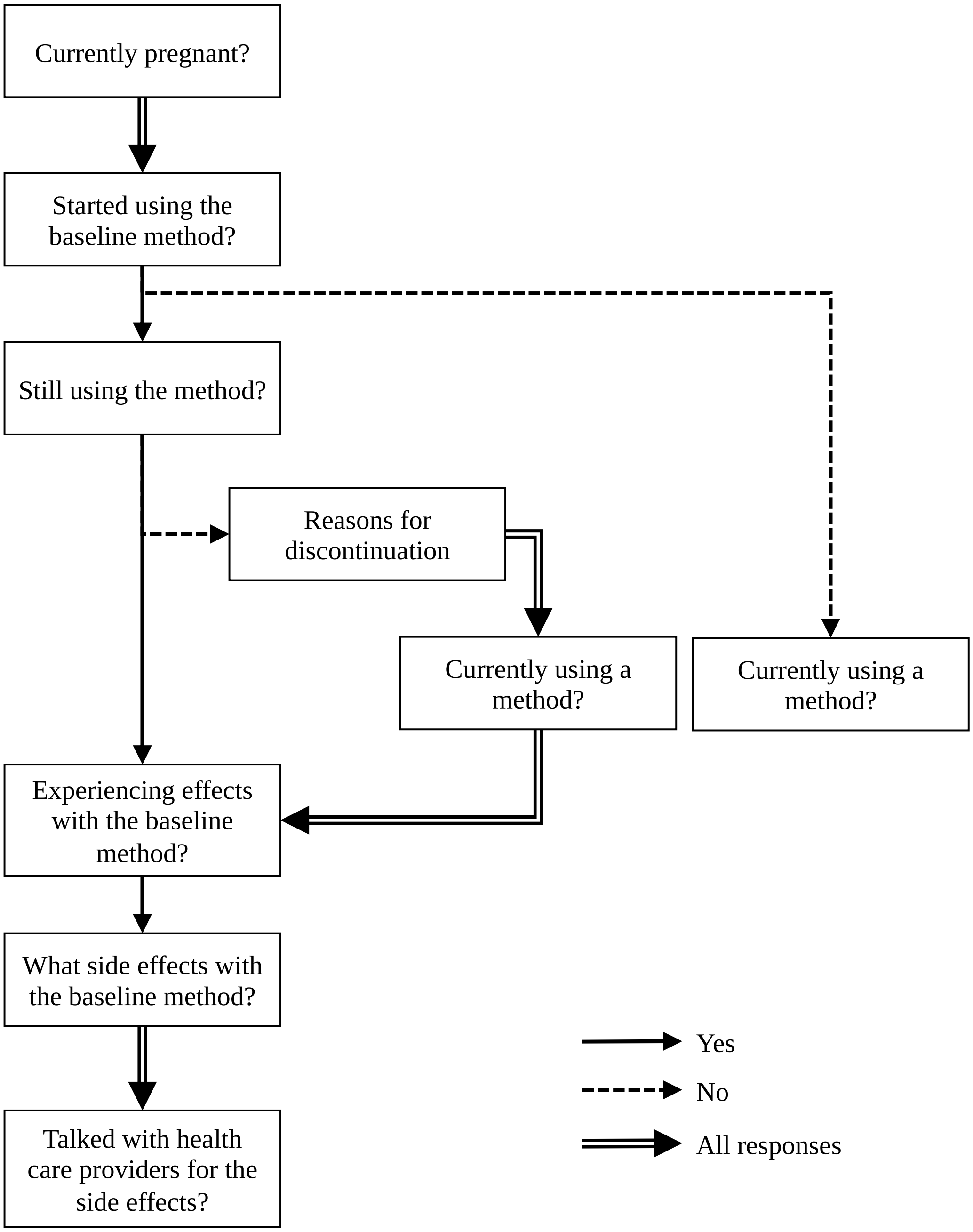

APPENDIX Table T2 Among women who received a prescription or method at baseline, adjusted associations between women's baseline quality item scores and reproductive outcomes at follow-up, by geography—results from multivariable regression models

	Burkina Faso (n=597)	Kenya (n=3,270)	Kano, Nigeria (n=479)	Lagos, Nigeria (n=346)
**Model and Reproductive Outcome**				
**Model 1: Continued protection from unintended pregnancy**	**aOR (95% CI)**			
Informational and interpersonal items only	1.25 (0.63 – 2.49)	1.12 (0.75 – 1.67)	2.00 (0.66 – 6.09)	1.45 (0.41 – 5.14)
Disrespect and abuse items only	1.19 (0.58 – 2.43)	0.88 (0.622 – 1.24)	0.93 (0.53 – 1.65)	0.75 (0.25 – 2.19)
**Model 2: Informational needs met**	**aβ (95% CI)**			
Informational and interpersonal items only	0.92*** (0.55 – 1.29)	1.09*** (0.88 – 1.30)	-0.28 (-1.09– 0.54)	-0.45 (-1.19 – 0.28)
Disrespect and abuse items only	0.01 (-0.38 – 0.38)	-0.25*** (-0.42 - -0.07)	-0.63*** (-1.04 - -0.21)	0.48 (-0.13 – 1.08)
**Model 3: Care-seeking for side effects^**	**aOR (95% CI)**			
Informational and interpersonal items only	1.21 (0.76 - 1.94)	1.35** (1.06 - 1.71)	2.36 (0.95 – 5.85)	0.85 (0.38 - 1.90)
Disrespect and abuse items only	0.78 (0.48 – 1.26)	0.95 (0.78 – 1.16)	0.98 (0.63 – 1.52)	1.02 (0.55 – 1.90)

Informational exchange and interpersonal items only (8 items); disrespect and abuse items only (2 items). Models adjusted for age, educational attainment, marital status, prior contraceptive method use. Marital status not included in Nigeria, due to small variation. Ref=reference group. aOR=adjusted odds ratio. aβ=adjusted linear regression coefficient. *** p<0.01, ** p<0.05, * p<0.1. Estimates adjusted for lost-to-follow-up analytical weights. ^Analyses restricted to women who with any side effects of their baseline method.
